# Manipulating avatar age and gender in level-2 visual perspective taking

**DOI:** 10.3758/s13423-023-02249-7

**Published:** 2023-02-13

**Authors:** B. Ford, R. Monk, D. Litchfield, A. Qureshi

**Affiliations:** Department of Psychology, Edge Hill University, Ormskirk, Lancashire UK

**Keywords:** Visual perspective taking, Avatar characteristics, Social cues, Spatial processing

## Abstract

Visual perspective taking (VPT) represents how the world appears from another person’s position. The age, group status and emotional displays of the other person have been shown to affect task performance, but tasks often confound social and spatial outcome measures by embedding perspective taking in explicitly social contexts or theory-of-mind reasoning. Furthermore, while previous research has suggested that visual perspective taking may be impacted by avatar characteristics, it is unknown whether this is driven by general group processing or a specific deficit in mentalizing about outgroups, for example, children. Therefore, using a minimally social task (i.e., the task was not communicative, and acknowledging the “mind” of the avatar was not necessitated), we examined whether avatar age and avatar gender affect performance on simpler (low angular disparity) and more effortful, embodied (high angular disparity) perspective judgments. Ninety-two participants represented the visuospatial perspectives of a boy, girl, man, or woman who were presented at various angular disparities. A target object was placed in front of the avatar and participants responded to the orientation of the object from the avatar’s position. The findings suggest that social features of visuospatial perspective taking (VSPT) are processed separately from the fundamental spatial computations. Further, Level-2 VSPT appears to be affected by general group categorization (e.g., age and gender) rather than a deficit in mentalizing about a specific outgroup (e.g., children).

**Supplementary Information:**

The online version contains supplementary material available at 10.3758/s13423-023-02249-7.

## Introduction

**Fig. 1:**
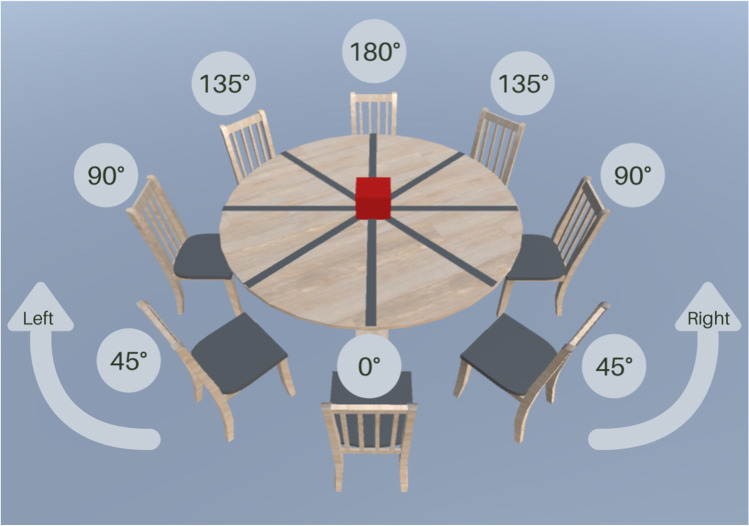
The image below demonstrates a typical manipulation of angular disparity. Here, the avatar’s position (depicted by an empty chair) rotates away from the shared line of sight at 0° along an imaginary arc that borders a jointly attended target object (red cube)

Representing another person’s visual perspective allows us to accurately communicate spatial information, generate shared frames of reference, and understand their knowledge of the world (Frith & Frith, 2007). Level-1 visual perspective taking (L1-VPT) involves tracking what another person can see, with the consistency of self and other perspectives determined by whether objects are jointly attended (Flavell et al., 1981; Qureshi et al., 2010). In L1-VPT, perspective calculation happens automatically (Qureshi & Monk, 2018; Samson et al., 2010; though see Heyes, 2014), whilst perspective selection occurs at a later stage of processing (McCleery et al., 2011) and requires inhibitory control (Qureshi et al., 2020). In contrast, Level-2 visuospatial perspective taking (L2-VSPT) represents *how* the world appears to another person, visually or spatially (Flavell et al., 1981). In L2-VSPT, an avatar who shares line-of-sight with the participant is situated at 0° and angular disparity increases with the avatar’s rotation away from this shared orientation (see Fig. 1). At low angular disparity (< 80°), Level-2 visual perspectives are relatively consistent, and participants can rely on their egocentric view (Wang et al., 2016). With increasing angular disparity comes greater cognitive demand, and posture congruency effects reveal an embodied mental self-rotation into the position of the “other” (Kessler & Thomson, 2010; Surtees et al., 2013a). This embodied self-rotation recruits neural regions involved in processing socially relevant stimuli, body schema, and executive function (Seymour et al., 2018).

Recent evidence indicates that social features, such as group-dynamics or agent emotions, impact L1-VPT. Savitsky et al. (2011) and Simpson and Todd (2017) had participants represent perspectives of in-group and out-group others. They postulate that an assumption of shared knowledge accompanying in-group categorization produces egocentric interference, which increases the effort required to understand inconsistent perspectives of ingroup members or friends compared to outgroup members or strangers. However, when perspectives are consistent, similarity or closeness is facilitative. Furthermore, Monk et al. (2020) studied VPT in the context of desire reasoning and reported that an agent’s emotional facial expression interacts with knowledge about their likes and dislikes to impact perspective representation. Taken together, findings from L1-VPT suggest that social cues modulate performance in simpler forms of VPT.

Similar social manipulations in the more controlled process of L2-VSPT have reported mixed results. Todd et al. (2011) found that maze completion took significantly longer when guiding an ingroup member, suggesting that less egocentric interference occurs with outgroup members, allowing them to understand inconsistent perspectives more easily. Conversely, using minimal groups,^1^ Ye et al. (2020) found facilitative effects for ingroup members, suggesting that dehumanization accompanying outgroup classification interfered with effective perspective representation. As Ye et al. (2020) note, differing task demands may explain these incongruent findings. Todd et al. (2011) involved mutual perspective taking where a guide represents the walker’s perspective and the walker interprets cues, whilst Ye and colleagues simply had participants represent perspectives of computerised avatars. As such, there is at least diminutive evidence that group categorization may impact L2-VSPT.

Nevertheless, when VSPT is embedded in social scenarios like desire reasoning (Monk et al., 2020), spatial navigation (Todd et al., 2011), or group categorization (Savitsky et al., 2011; Simpson & Todd, 2017; Ye et al., 2020), it remains unclear whether social cue processing is characteristic of perspective computation or relevant only when perspective taking occurs in a communicative or social context. Clements-Stephens et al. (2013) hypothesize that VSPT is predominantly spatial irrespective of the positional-cue (e.g., human or arrow), but becomes increasingly social with agent-like cues and additional socially relevant knowledge. For instance, spatial cues facilitate performance on spatial orientation tasks specifically when they also contain agentic information such as a human figure (Geer & Ganley, 2022; Gunalp et al., 2019). Crucially, as participants are not required to mentalize or explicitly acknowledge social distinctions, these tasks directly examine the effect of “social” cues on spatial perspective processing. Thus, manipulations of socially relevant features need not be explicit, as the social function of real-world socio-cognitive processing regularly takes place in suboptimal circumstances (Mussweiler, 2003). Individuals often make social evaluations under time pressure and whilst possessing little knowledge of the target (Mussweiler & Strack, 1999), promoting the use of accessible physical characteristics (Mussweiler, 2003) such as gender and age (Cloutier et al., 2005; Cloutier et al., 2014; Macrae & Bodenhausen, 2000). Therefore, social cue effects need to be examined in a task that does not prime participants to focus on distinctive features, but rather presents an implicit examination by manipulating physically observable characteristics of social groups such as age and gender.

Recently advancing our understanding of social cues in VSPT, Ward et al. (2020) manipulated avatar gaze in a minimally social L2-VSPT task. The authors argued that if social cues are important for L2-VSPT, whether the other’s gaze is fixated on the stimulus should be integrated into task processing. Results showed that participants spontaneously incorporated knowledge of the other’s position regardless of whether their gaze was directed towards the object. Emphasizing the spatial processes at the heart of VSPT, Ward et al. (2020) concluded that the other person in L2-VSPT tasks functions as a spatial landmark rather than “eyes to peer through,” minimizing the importance of even perceptually relevant social cues.

Yet, avatar age does affect L1-VPT. Ferguson et al. (2018) used a minimally social task and found that adults did not automatically compute the perspectives of children as they do for adults, with significantly weaker interference from an irrelevant child’s perspective when answering from an egocentric view. Ferguson et al. (2018) provided two competing explanations for this apparently reduced interference. First, adults do not preferentially select a child’s gaze as they do for other adults because of an own-age bias. Alternatively, given the long developmental trajectory of theory-of-mind, adults may assume a reduced mental capacity for children and, thus, children’s perspectives are less salient. It therefore remains to be examined whether general group processes or a diminished theory-of-mind for children, or indeed other outgroup members, modulates perspective representation. Evidence that social comparison processes modulate L1-VPT performance (Ferguson et al., 2018), but perceptually relevant gaze cues do not in L2-VSPT (Ward et al., 2020), raises questions about the interaction between social features and cognitive demand. Yet, there has been no previous research to explore this within L2 VPT.

Using a minimally social task, the current research therefore examines whether avatar age or gender differentially affects simple perspective processing (low angular disparity judgments) or more controlled perspective processing (high angular disparity judgments). Specifically, it will illuminate social and perceptual processing integration, explore the limits of avatar-characteristic influence in L2-VSPT, and determine whether general group processing or age-dependent mentalizing explains social processing effects in L2-VSPT. Gender and age were manipulated for three reasons. First, gender and age are easily accessible physical characteristics (Cloutier et al., 2014; Macrae & Bodenhausen, 2000) of day-to-day social relevance, rather than arbitrary or temporary group-defining characteristics (Ferguson et al., 2018). Second, rather than priming attention towards distinctive features, the current study measures implicit processing of social characteristics (Geer & Ganley, 2022; Gunalp et al., 2019; Gunalp et al., 2021). Third, manipulating gender *and* age helps distinguish the diminished awareness for children’s minds and general group-processing hypotheses (Ferguson et al., 2018). Participants completed a L2-VSPT task and made left/right judgments about an object’s orientation from the perspective of male and female adults and children sat at varying angular rotations (45°/90°/135°).

The following hypotheses were assessed and are summarized in Table 1:

1. Replicating previous findings (Kessler & Rutherford, 2010; Kessler & Thomson, 2010; Michelon & Zacks, 2006; Surtees et al., 2013a), it is hypothesized that participants will be significantly quicker, and more accurate, at 45° compared to 90°, and at 90° compared to 135°.

**Table 1:** Summary of exploratory hypotheses

IVs	Research question	Hypothesis	Possible results
Across all angles	Can an online testing procedure replicate previous work that has found slowing response times and decreasing accuracy as angular disparity increases?	RT will increase with increasing angular disparity Accuracy will decrease with increasing angular disparity	RTs will be significantly longer at 90° than 45°, and significantly longer at 135° than 90° Accuracy will be significantly lower at 90° than 45°, and significantly lower at 135° than 90°
Low angular disparity (45°)	Is social categorization apparent in simple perceptual tasks?	RT will be quicker if similarity between self and other is facilitative	RT for same gender adult avatars will be quicker than all other avatars. The difference between RTs of other avatars will not differ significantly
	Is mental state attribution apparent in simple perceptual tasks?	RTs for child avatars will be slower if we do not as readily compute their visual perspectives / mental representations	Fixed effect of age but not gender
Higher angular disparity (90° and 135°)	Is there evidence for mental state attribution during L2-VSPT?	RT for child avatars will be slower if we do not as readily compute their perspectives	Fixed effect of age but not gender
	Is there evidence for social comparison processes exerting top-down control over L2-VSPT?	RT differences between same-gender-adult avatars and the other avatars	1) RT for similar avatar could be quicker if facilitative 2) RT for similar avatars could be slower if egocentric interference causes interference

*RT* response time, *L2-VSPT* Level-2 visuospatial perspective taking

Given the paucity of research, analyses regarding the interaction between agent characteristics and VSPT are exploratory for low and high angular disparity:

2. At low angular disparity (45°), if general group processes exert influence over visuospatial processing, similarity may be facilitative (Savitsky et al., 2011; Simpson & Todd, 2017) and response times for same-gender adult avatars will be significantly quicker than for dissimilar avatars. Response times between other avatars, all being dissimilar to the self in at least one characteristic, are not expected to differ significantly. Alternatively, if we assume a reduced mental capacity for children (Ferguson et al., 2018), we expect a significant effect of avatar age, regardless of gender, evidenced by slower response times for child avatars.
3. At higher angles (90° and 135°), if general group processes exert influence over visuospatial processing, significantly different response times are hypothesized between same-gender adult avatars and others. Replicating previous findings (Savitsky et al., 2011; Simpson & Todd, 2017; Todd et al., 2011), greater egocentric interference for same-gender adult avatars may result in slower response times compared to dissimilar avatars. However, it is also possible that avatar dissimilarity could cause interference that increases response times compared to same-gender adult avatars (akin to Ye et al., 2020). Alternatively, if we assume a reduced mental capacity for children (Ferguson et al., 2018), irrespective of gender, we expect significantly slower response times for child avatars.

## Method

### Participants

For linear mixed effects models, to discover an effect analogous to *d* = .25 with a power of .75 and 288 stimuli, a minimum sample size of 85 participants is required (Westfall et al., 2014). Acknowledging that online studies may produce noisier data (Bridges et al., 2020) and risk poorer data quality, 99 participants (35 male; 62 female; one non-binary; one undeclared) with a mean age of 29.95 (*SD* = 8.76) were recruited online via opportunity sampling (n = 39) and Prolific Academic (n = 60) and received £3.00 remuneration for ~20 min of their time. The experiment was granted ethical approval by the Departmental Research Ethics Committee. Participants that did not finish the task (n = 2), did not complete demographic information (n = 1), did not meet eligibility criteria (n = 1), identified as a gender other than male or female (n = 1), or had < 66% accuracy (n = 2) were excluded from further analysis. This left a total of 92 participants (34 men, 58 women; *M*_age_ = 30.22, *SD*_age_ = 8.91, range = 18–63) whose data was able to be included in the analysis.

### Stimuli

**Fig. 2:**
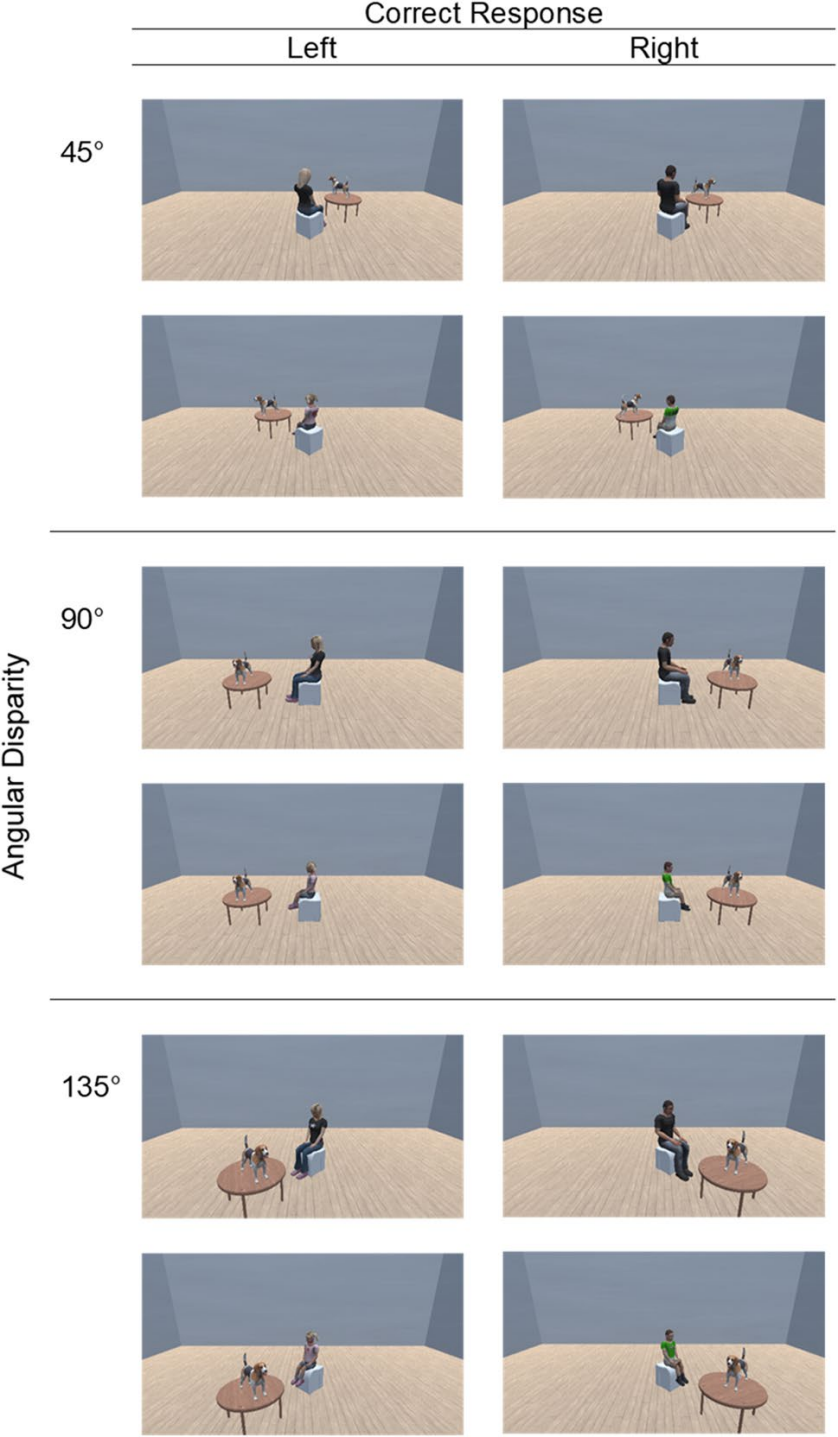
Example stimuli including female adults and children (left column) and male adults and children (right column). Each row of four pictures represents a specific angular disparity (left-rotation direction placed above right-rotation directions). Correct responses to object direction also specified with “Left” responses in the left-hand column and “Right” responses in the right-hand column

Stimuli were a series of images containing an avatar (female child/female adult/male child/male adult) sitting in front of, and looking at, a dog. Experimental stimuli are based on Surtees et al. (2013a) but with realistic-looking avatars (Kessler & Rutherford, 2010; Kessler & Thomson, 2010; Ye et al., 2020). Realistic stimuli were important for the distinction between males, females, adults, and children. Angular disparity was determined by the rotation of the avatar in relation to the viewing position of the participant. Example stimuli are shown in Fig. 2. A dog was chosen because the direction the dog is facing is easily determined from spatial cues (for use of a similar focus object, see Hamilton et al., 2009). The stimuli were designed in the Unity game engine (Unity Technologies, 2020) and the experiment was programmed using PsychoPy3 (Peirce et al., 2019) and run on Pavlovia.org (Bridges et al., 2020). Original stimuli dimensions were 1,920 × 1 080. On-screen, stimuli height was set at 90% of the participant’s window and width was set at 1.78 × height.

### Design

The study used a 2 × 2 × 3 repeated-measures design with avatar age (adult/child), avatar gender (same/other), and angular disparity (45°/90°/135°) as factors. To code the avatar gender, participants who identified themselves as female had “woman” and “girl” stimuli recoded to “same-gender” and “man” and “boy” stimuli recoded to “other-gender,” and vice versa. To provide the greatest opportunity for social processing, the decision was made to position the avatar centrally for maximum saliency (Fig. 1). The avatars were rotated at 45°, 90°, or 135° to the left or right, which was later collapsed across left/right for fixed-effects analysis; original stimuli information was retained for inclusion as a random effect.

### Procedure

Participants were provided with study information and informed consent on Qualtrics (Qualtrics, Provo, UT, USA) and, after agreeing to participate, they were redirected to the task on Pavlovia.org (Bridges et al., 2020). Participants completed 12 practice trials (one for each condition) before completing 288 experimental trials split across six blocks of 48 trials (24 trials per condition). Participants were instructed to take the avatar’s perspective on all trials and make spatial (left or right) judgments about a dog’s orientation using the corresponding directional key. Specifically, participants were told that they will be presented with images of avatars looking at a dog standing on a table and that they must respond to the question “From the avatar’s view, which direction is the dog facing?” This instruction was presented at the beginning of the experiment and restated during the breaks. Participants were instructed to press ‘Q’ if the dog was facing left, or ‘P’ if the dog was facing right. On each trial a fixation cross was presented for a randomised duration between 1,500 and 2,500 ms. The experimental stimuli were then presented until the participant made a response. In total, the task took ~20 min to complete.

### Online data quality

To facilitate good quality data, participants recruited from Prolific Academic had declared themselves as fluent speakers of English, completed at least ten prior studies, and had 100% approval ratings. The number of trials per condition was maximized to account for the expected variability from collecting data online whilst keeping block duration (~3 min) and overall participation time (~20 min) brief to facilitate attention (Sauter et al., 2020). Mean accuracy was high in all conditions (all >.95) and only two participants were removed for having accuracy below 66%. Trial loss because of response times ±2.5 SD than individual condition means was 5.83%, with similar lab-based studies reporting analogous data loss of 3.1% (Surtees et al., 2013a), or between 2.81% and 3.65%, depending on condition (Surtees et al., 2013b).

## Results

Analyses were conducted using lme4 (Bates et al., 2015) for R (R Core Team, 2017). Factors of angle (45°/90°/135°), avatar gender (man/woman) and age (child/adult) were entered into a linear mixed effects model for response times and mixed-effect logistic regression for accuracy. Random intercepts were included to control for variation across individuals and stimuli, reducing the potential for type 1 error that can arise if they are treated as fixed (Barr, 2013; Barr et al., 2013).^2^ Including random slopes for condition effects (as per Barr et al., 2013) led to model non-convergence and, thus, they were not included in the final models. See Online Supplementary Materials (OSM) for model comparisons. As comparing low angular disparity (45°) to higher angles (90° and 135°) was of interest, angle was treatment coded to compare each higher angle to the baseline of 45°. Alternatively, for age and gender variables with only two levels, contrast coding was used.

### Accuracy

**Fig. 3:**
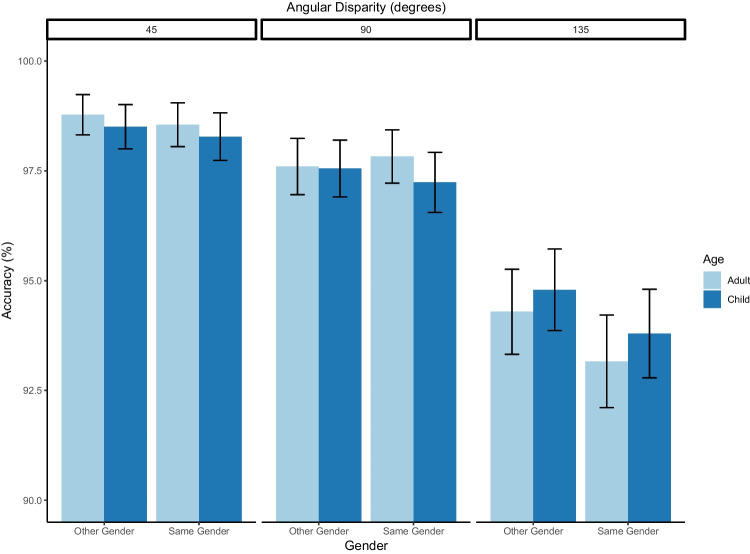
Accuracy rates by angle (45/90/135°), avatar age (Adult/Child) and avatar gender (Same/Other). Error bars = confidence intervals

Analyses found a fixed effect of 90° approaching the typical significance threshold (*OR* = 0.57, *p* = .05) and a significant fixed effect of 135° (*OR* = 0.15, *p* < .001). Further analysis confirmed significantly less accuracy at 135° (*M* = 0.95, *SD* = 0.21) compared to 90°, *OR* = 5.24, *p* < .001. No other fixed effects or interactions were significant (all *p*s > .19; see Table 2 and Fig. 3 for full results).

**Table 2:** Mixed effect logistic regression on accuracy data for angle × avatar age × avatar gender

	Odds ratio	95% CI	*p*
Fixed effect: Angle			
Baseline: 45°			
90°	0.57	0.31–1.02	0.05
135°	0.15	0.11–0.24	<0.001
Fixed effect: Avatar age			
Baseline: Adult			
Child	0.80	0.38–1.53	0.48
Fixed effect: Avatar gender			
Baseline: Same gender			
Other gender	1.05	0.56–1.97	0.87
Interaction: Angle × Avatar age			
Baseline: 45°/Child			
90° × Adult	1.13	0.51–2.51	0.76
135° × Adult	1.58	0.78–3.28	0.19
Interaction: Angle × Avatar gender			
Baseline: 45°/Same gender			
90° × Other gender	0.92	0.42–2.02	0.82
135° × Other gender	1.25	1.09–1.93	0.52
Interaction: Avatar age × Avatar gender			
Baseline: Adult/Same gender			
Child × Other gender	1.19	0.50–2.83	0.69
Interaction: Angle × Avatar age × Avatar gender			
Baseline: 45°/Adult/Same gender			
90° × Child × Other-gender	0.88	0.29–2.61	0.81
135° × Child × Other-gender	0.74	0.28–1.94	0.52
(Intercept)	214.15	127.35–382.07	<0.001

### Response times (accurate trials only)

**Fig. 4:**
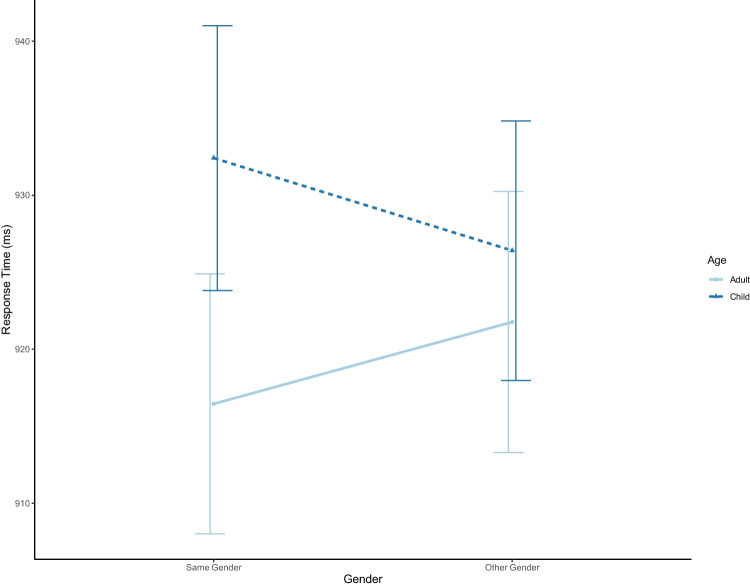
Line plot for the interaction between avatar age (Adult/Child) and avatar gender (Same/Other) collapsed across angles. Error bars = confidence intervals

Analyses found significant fixed effects of 90° (*β* = .08, *t* = 4.21, *p* < .001) and 135° (*β* = 0.28, *t* = 15.36, *p* < .001). No fixed effects of age or gender reached significance (both *p*s > .07). However, there was a significant interaction between age and gender, *β* = -0.02, *t* = -2.06, *p* = .04. Figure 4 shows the interaction is likely driven by slower response times for child versus adult avatars when they are the same gender as participants. However, coefficient confidence intervals cross zero so the interaction should be interpreted cautiously. No other interactions reached significance (see Table 3 for full results).

**Table 3:** Linear mixed effects model on response times for Angle × Avatar Age × Avatar Gender

	Coefficients	95% CI	*t*-value	*p*
Fixed effect: Angle				
Baseline: 45°				
90°	0.08	0.04–0.11	4.21	<0.001
135°	0.28	0.24–0.31	15.36	<0.001
Fixed effect: Avatar age				
Baseline: Adult				
Child	0.02	-0.01–0.06	1.34	0.18
Fixed effect: Avatar gender				
Baseline: Same gender				
Other gender	0.02	-0.001–0.03	1.80	0.07
Interaction: Angle × Avatar age				
Baseline: 45°/Child				
90° × Adult	-0.01	-0.06–0.04	-0.42	0.67
135° × Adult	-0.01	-0.07–0.04	-0.58	0.57
Interaction: Angle × Avatar gender				
Baseline: 45°/Same gender				
90° × Other gender	-0.01	-0.03–0.01	-0.75	0.45
135° × Other gender	-0.02	-0.05–0.001	-1.82	0.07
Interaction: Avatar age × Avatar gender				
Baseline: Adult/Same gender				
Child × Other gender	-0.02	-0.05–0.001	-2.06	0.04
Interaction: Angle × Avatar age × Avatar gender				
Baseline: 45°/Adult /Same gender				
90° × Child × Other gender	0.01	-0.02–0.04	0.58	0.56
135° × Child × Other gender	0.03	-0.001–0.06	1.85	0.06
(Intercept)	0.80	0.75–0.85	34.73	<0.001
Random effects	Estimate	SD		
Participant	0.03	0.19		
Stimulus	<0.01	0.03		
Residual	0.07	0.26		

## Discussion

Previous research suggests that social features affect VSPT task performance, but confounded measures mean it is difficult to disentangle whether social cues impact fundamental perspective computation or the communicative and social elements of tasks. Furthermore, despite growing emphasis on agent characteristics in L1-VPT (visibility judgments), little attention has been given to L2-VSPT *(how* the world appears)*.* Accordingly, this study is the first to use a minimally social task to explore the effects of agent characteristics in L2-VSPT. Results suggest that implicit processing of avatar age modulates response times only for same-gender avatars. Explanations address how social processing may be incorporated into L2-VSPT, provide support for the general group processing hypothesis rather than a diminished salience of children’s minds, and help determine the limits of avatar-characteristic effects.

The significant fixed effect of angle found for accuracy and response times replicates a considerable amount of previous work that suggests that cognitive effort increases with increasing angular disparity and, thus, accuracy decreases, and response times increase (Kessler & Rutherford, 2010; Kessler & Thomson, 2010; Michelon & Zacks, 2006; Surtees et al., 2013a). Replicating well-established findings suggests adequate sensitivity to detect experimental effects in an online task, increasing confidence that Type 2 errors are unlikely.

To understand how social processing integrates with VSPT, it was hypothesized that avatar characteristics may have differentially affected the distinct processing at lower and higher angular disparities. At low angles, participants can represent an agent’s visual perspectives using their egocentric reference frame (Wang et al., 2016), but high angular disparity judgments involve a mental self-rotation into the other’s position (Kessler & Thomson, 2010). Clements-Stephens et al. (2013) proposed that the initial processing in L2-VSPT involves embodying the spatial position, with consideration of social attributes coming after. The cross-angle age-gender interaction reported indicates that implicit processing of defining physical characteristics exerts influence regardless of perspective representation strategy. The absence of differential effects is unsurprising if we consider that “others” in visuospatial tasks are predominantly used as landmarks to rotate the self-referential frame towards, rather than “seeing through their eyes” per se (Gunalp et al., 2019; Ward et al., 2020). Furthermore, the fundamental spatial processes key to VSPT may not be tightly integrated with networks incorporating socially relevant information, as these processes have only been repurposed for social functioning evolutionarily later (Kessler & Thomson, 2010; Ward et al., 2019). Therefore, supporting Clements-Stephens et al.’s (2013) proposition, our cross-angle interaction may indicate that only once the fundamental spatial processing is complete does social processing exert its limited effect.

Regarding the specific social processing occurring during perspective representation, Ferguson et al. (2018) reported two competing explanations for age-of-avatar effects in L1-VPT: either the saliency of children’s perspectives was reduced by general group categorization or adults assume a diminished mental capacity for children. Had gender not been manipulated, the slower response times reported for same-gender children could have supported the diminished mental capacity hypothesis in L2-VSPT. However, as we found no analogous effect on response times for other-gender children, the results support a general group processing hypothesis.

It is important to note that our results represent more complex categorization processes than have been reported in previous literature where simple group categorization modulates interference or facilitation (Savitsky et al., 2011; Todd et al., 2017; Ye et al., 2020). If all avatars that were dissimilar to the self in at least one characteristic were simply categorized as “outgroup,” then we would expect similar patterns for other-gender avatars as for same-gender children, which we do not see. Although not significant, response times for other-gender adults and children were slower than same-gender adults but quicker than same-gender children. Speculatively, when adults perceived other-gender adults, we suggest that outgroup classification was immediately determined by gender, with age not being as functionally relevant (Cloutier et al., 2014; Fitousi, 2017). Alternatively, when adults perceived other-gender child avatars, out-group categorization began with age and was reinforced with gender (Cloutier et al., 2014). Using this rationale, same-gender children were classified immediately as “other” based on age, but the same-gender classification introduced greater complexity. Therefore, modulation of response times to same-gendered avatars was significant across angles because of marginal facilitation of similarity and marginal interference of categorization complexity, relative to simpler outgroup categorization. Considering the competing explanations from Ferguson et al. (2018), for L2-VSPT, effects of avatar characteristics derive from general group classification rather than an assumed reduced mental capacity for children.

The current findings also raise methodological questions for how best to measure social perspective taking. The absolute differences in response times between avatars were minimal and the effects of social cues, compared to spatial cues like angle, were small. Previous research that has found larger group-related effects embedded VSPT in social contexts that explicitly relate to the target agents (Todd et al., 2011; Ye et al., 2020). For example, communicative tasks (Savitsky et al., 2011) indicate a “mind to be known,” evoking theory-of-mind, and tasks pre-testing to allocate groups (Ye et al., 2020) suggest avatar similarity and differences are important considerations. Furthermore, Tarampi et al. (2016) found that framing spatial orientation tasks as measuring empathy instead of spatial skills significantly improved female performance. Thus, our results suggest that when visuospatial representation is embedded in a social context, the salience of social cues is amplified, increasing the influence of group processes over perspective representation. This highlights the usefulness of minimally social tasks to understand how isolated social variables interact with visuospatial computations (Ferguson et al., 2018; Geer & Ganley, 2022; Gunalp et al., 2021; Ward et al., 2020) and has important implications for the validity of social inferences depending on the inclusion of, and interaction between, social features in L2-VSPT tasks.

## Limitations

The spatial responses required may have primed participants towards spatial processing. Imagining a contrasting visual scene may instead focus attention on “seeing through their eyes.” Future research could explore whether avatar characteristics affect visual and spatial perspective taking differently (see Surtees et al., 2013b). Furthermore, the current task could not examine how avatar characteristics affected egocentric judgments as the interaction between social processing and angular disparity was of particular interest. Pragmatically, it was imperative to keep task duration short, retain an adequate number of stimuli per condition, and not further complicate the factor structure. A task that permits “self-versus-other” contrasts could further our understanding of spontaneous perspective taking in Level-1 (Ferguson et al., 2018) and Level-2 (Ward et al., 2019). As is typical in VPT, low error rates meant accuracy analysis was of little inferential use (Michelon & Zacks, 2006; Surtees et al., 2013a, 2013b). Future tasks could increase complexity by including unfamiliar objects, more orientations, or both. Moreover, considering large effects may be robust to increased response time variability in online testing but effects of social manipulations are small, lab-based research using implicit measurements such as eye-tracking would be useful.

## Conclusion

The current findings offer three important insights. First, they provide further evidence that social features in VSPT may be processed separately from fundamental spatial computations. Second, they indicate that general group categorization, rather than diminished mentalizing about children, affects L2-VSPT. Third, they highlight the significant role of context in amplifying social processing effects. These insights suggest that despite the fundamentally spatial computations required, while social context magnifies the salience of social cues, implicit processing of group-defining characteristics occurs even during minimally social perspective representation.

## Supplementary information

ESM 1 (DOCX 89.5 kb)

## Footnotes

1 Minimal group designs attempt to create in-group out-group dynamics from trivial distinctions such as colored badges or fake results from a task-irrelevant pre-test (Diehl, 1990).

## References

[CR1] Barr DJ (2013). Random effects structure for testing interactions in linear mixed-effects models. Frontiers in Psychology.

[CR2] Barr DJ, Levy R, Scheepers C, Tily HJ (2013). Random effects structure for confirmatory hypothesis testing: Keep it maximal. Journal of Memory and Language.

[CR3] Bates, D., Kliegl, R., Vasishth, S., & Baayen, H. (2015). Parsimonious mixed models.

[CR4] Bridges D, Pitiot A, MacAskill MR, Peirce JW (2020). The timing mega-study: Comparing a range of experiment generators, both lab-based and online. PeerJ.

[CR5] Clements-Stephens AM, Vasiljevic K, Murray AJ, Shelton AL (2013). The role of potential agents in making spatial perspective taking social. Frontiers in Human Neuroscience.

[CR6] Cloutier J, Mason MF, Macrae CN (2005). The perceptual determinants of person construal: Reopening the social-cognitive toolbox. Journal of Personality and Social Psychology.

[CR7] Cloutier J, Freeman JB, Ambady N (2014). Investigating the early stages of person perception: The asymmetry of social categorization by sex vs. age. PLoS ONE.

[CR8] Diehl M (1990). The minimal group paradigm: Theoretical explanations and empirical findings. European Review of Social Psychology.

[CR9] Ferguson HJ, Brunsdon VEA, Bradford EEF (2018). Age of avatar modulates the altercentric bias in a visual perspective-taking task: ERP and behavioral evidence. Cognitive, Affective, & Behavioral Neuroscience.

[CR10] Fitousi D (2017). Binding sex, age, and race in unfamiliar faces: The formation of “face files”. Journal of Experimental Social Psychology.

[CR11] Flavell JH, Everett BA, Croft K, Flavell ER (1981). Young children's knowledge about visual perception: Further evidence for the Level 1–Level 2 distinction. Developmental Psychology.

[CR12] Frith CD, Frith U (2007). Social cognition in humans. Current Biology.

[CR13] Geer, E. A., & Ganley, C. (2022). Sex Differences in Social and Spatial Perspective Taking: A Replication and Extension of Tarampi et al. (2016). *Quarterly Journal of Experimental Psychology*, 17470218221085117. 10.1177/17470218221085117 35179057

[CR14] Gunalp P, Moossaian T, Hegarty M (2019). Spatial perspective taking: Effects of social, directional, and interactive cues. Memory & Cognition.

[CR15] Gunalp P, Chrastil ER, Hegarty M (2021). Directionality eclipses agency: How both directional and social cues improve spatial perspective taking. Psychonomic Bulletin & Review.

[CR16] Hamilton AFDC, Brindley R, Frith U (2009). Visual perspective taking impairment in children with autistic spectrum disorder. Cognition.

[CR17] Heyes, C. (2014). False belief in infancy: a fresh look. *Developmental Science, 17*(5), 647–659. 10.1111/desc.12148 24666559

[CR18] Kessler K, Rutherford H (2010). The two forms of visuo-spatial perspective taking are differently embodied and subserve different spatial prepositions. Frontiers in Psychology.

[CR19] Kessler K, Thomson LA (2010). The embodied nature of spatial perspective taking: Embodied transformation versus sensorimotor interference. Cognition.

[CR20] Macrae CN, Bodenhausen GV (2000). Social cognition: Thinking categorically about others. Annual Review of Psychology.

[CR21] McCleery JP, Surtees ADR, Graham KA, Richards JE, Apperly IA (2011). The Neural and Cognitive Time Course of Theory of Mind. Journal of Neuroscience.

[CR22] Michelon P, Zacks JM (2006). Two kinds of visual perspective taking. Perception & Psychophysics.

[CR23] Monk RL, Colbert L, Darker G, Cowling J, Jones B, Qureshi AW (2020). Emotion and liking: How director emotional expression and knowledge of (dis)liking may impact adults’ ability to follow the instructions of an ignorant speaker. Psychological Research.

[CR24] Mussweiler T (2003). Comparison processes in social judgment: Mechanisms and consequences. Psychological Review.

[CR25] Mussweiler T, Strack F (1999). Hypothesis-consistent testing and semantic priming in the anchoring paradigm: A selective accessibility model. Journal of Experimental Social Psychology.

[CR26] Peirce, J., Gray, J. R., Simpson, S., MacAskill, M., Höchenberger, R., Sogo, H., ... & Lindeløv, J. K. (2019). PsychoPy2: Experiments in behavior made easy. *Behavior Research Methods*, *51*(1), 195–203. 10.3758/s13428-018-01193-y PMC6420413 30734206

[CR27] Qureshi, A. W., & Monk, R. L. (2018). Executive function underlies both perspective selection and calculation in Level-1 visual perspective taking. *Psychonomic Bulletin & Review, 25*(4), 1526–1534. 10.3758/s13423-018-1496-8 PMC6096641 29949017

[CR28] Qureshi AW, Apperly IA, Samson D (2010). Executive function is necessary for perspective selection, not Level-1 visual perspective calculation: Evidence from a dual-task study of adults. Cognition.

[CR29] Qureshi, A. W., Monk, R. L., Samson, D., & Apperly, I. A. (2020). Does interference between self and other perspectives in theory of mind tasks reflect a common underlying process? Evidence from individual differences in theory of mind and inhibitory control. *Psychonomic Bulletin & Review, 27*(1), 178–190. 10.3758/s13423-019-01656-z PMC7000534 31429057

[CR30] R Core Team. (2017). R (Version 4.1.2) [computer software]. https://cran.r-project.org/bin/windows/base/

[CR31] Samson D, Apperly IA, Braithwaite JJ, Andrews BJ, Bodley Scott SE (2010). Seeing it their way: Evidence for rapid and involuntary computation of what other people see. Journal of Experimental Psychology: Human Perception and Performance.

[CR32] Sauter M, Draschkow D, Mack W (2020). Building, hosting and recruiting: A brief introduction to running behavioral experiments online. Brain Sciences.

[CR33] Savitsky K, Keysar B, Epley N, Carter T, Swanson A (2011). The closeness-communication bias: Increased egocentrism among friends versus strangers. Journal of Experimental Social Psychology.

[CR34] Seymour, R.A., Wang, H., Rippon, G., & Kessler, K. (2018). Oscillatory networks of high-level mental alignment: A perspective-taking MEG study. *NeuroImage, 177*, 98–107. 10.1016/j.neuroimage.2018.05.016 29746907

[CR35] Simpson AJ, Todd AR (2017). Intergroup visual perspective-taking: Shared group membership impairs self-perspective inhibition but may facilitate perspective calculation. Cognition.

[CR36] Surtees A, Apperly I, Samson D (2013). The use of embodied self-rotation for visual and spatial perspective-taking. Frontiers in Human Neuroscience.

[CR37] Surtees A, Apperly I, Samson D (2013). Similarities and differences in visual and spatial perspective-taking processes. Cognition.

[CR38] Tarampi MR, Heydari N, Hegarty M (2016). A tale of two types of perspective taking: Sex differences in spatial ability. Psychological Science.

[CR39] Todd AR, Hanko K, Galinsky AD, Mussweiler T (2011). When focusing on differences leads to similar perspectives. Psychological Science.

[CR40] Todd, A. R., Cameron, C. D., & Simpson, A. J. (2017). Dissociating processes underlying level-1 visual perspective taking in adults. *Cognition, 159*, 97–101. 10.1016/j.cognition.2016.11.010 27915132

[CR41] Unity Technologies. (2020). Unity (Version 2021.1.0) [computer software]. https://unity.com/releases/editor/archive

[CR42] Wang H, Callaghan E, Gooding-Williams G, McAllister C, Kessler K (2016). Rhythm makes the world go round: An MEG-TMS study on the role of right TPJ theta oscillations in embodied perspective taking. Cortex.

[CR43] Ward E, Ganis G, Bach P (2019). Spontaneous vicarious perception of the content of another’s visual perspective. Current Biology.

[CR44] Ward E, Ganis G, McDonough KL, Bach P (2020). Perspective taking as virtual navigation? Perceptual simulation of what others see reflects their location in space but not their gaze. Cognition.

[CR45] Westfall J, Kenny DA, Judd CM (2014). Statistical power and optimal design in experiments in which samples of participants respond to samples of stimuli. Journal of Experimental Psychology: General.

[CR46] Ye T, Furumi F, Catarino da Silva D, Hamilton A (2020). Taking the perspectives of many people: Humanization matters. Psychonomic Bulletin & Review.

