# Supplementary material for: Manipulating avatar age and gender in level-2 visual perspective taking
Source: Psychon Bull Rev. 2023 Feb 13;30(4):1431–41. doi: 10.3758/s13423-023-02249-7 (PMC10482764; doi:10.3758/s13423-023-02249-7)
Supplement: Supplementary file 1 — (DOCX 89.5 kb) [file 13423_2023_2249_MOESM1_ESM.docx]

**Supplementary Material**

**Analysis**

***Pre-processing and mixed-effect model rationale***

Data were pre-processed in R (R Core Team, 2020). Trials with response times ±2.5SD from the participant’s condition mean were excluded, as well as inaccurate trials being excluded for response time analysis. A linear mixed effects model was conducted for response times (accurate trials only) and a mixed-effect logistic regression for accuracy. The normal approximation to the t-distribution was used to determine the significance (Barr et al., 2013). Mixed models were chosen for several reasons: improved statistical power, which was desirable considering the online data collection method; they do not assume independence like repeated measures ANOVA; participant and stimuli can be entered into the model as random effects; they reduce artificial sample-size inflation and the likelihood of type-1 error; and they are robust to missing data (e.g. errors and outliers), which was desirable considering the online data collection method (Baayen et al., 2008). Accordingly, random intercepts for participant and stimulus were entered into the mixed effects models to control for variation across individuals and stimuli, which reduces the potential for type 1 error that can arise if they are treated as fixed (Barr, 2013; Barr et al., 2013)^2^. This was also beneficial considering the limited control over the computer, operating system and browser participants used to complete the task.

**Confirming the inclusion of stimuli and ID as random effects**

For response time analysis, model comparison confirmed their inclusion significantly improved model fit (both *p*s <.001) with Bayes factors indicating extreme level of support for each. For accuracy analysis, model comparison also confirmed that including participant as a random effect reached significance (*p* <.001) with a Bayes factor representing overwhelming evidence for its inclusion. Interestingly, although Bayes factor indicated very strong evidence for the inclusion of stimuli as a random effect, the *p*-value was not below the typically accepted alpha value of 0.05 (*p* = .11)^[[1]](#footnote-1)^.

**Three-way interaction approaching significance**

**Figure 5.**

*Line plot depicting the three-way interaction between angle, avatar-age angle, and avatar-gender. Error bars = confidence intervals.*

**Model comparisons for response time and accuracy data**

***Model Comparison***

Considering that it is not statistically or logically correct to assume the absence of an effect from a null result (Lakens, et al., 2020), it is important to compare models to select the most parsimonious and provide greater support for the inclusion or omission of specific variables.

**Accuracy**

Confirming the results of hypothesis testing for accuracy data, the most parsimonious model is one that includes only angle as a fixed factor, X² (2) =75.54, *p* <.001, pseudo-R² = .09, with a Bayes factor indicating overwhelming support. Comparing an intercept only model to a model with avatar-gender as a fixed factor produced a *p*-value of just above .05, though pseudo-*R²* revealed no further variance explained. Moreover, Bayesian information criterion derived Bayes factors did not suggest support for the avatar-gender only model but also only anecdotal support for the intercept only model. No interactions had any significant effect on model fit (all *p*s >.25) with Bayes factors representing moderate to extreme evidence for the comparison models. See Table 5 for full results.

**Response Times**

For response time data, compared to an intercept-only model, including a fixed effect factor of angle significantly improved model fit X² (2) =117.18, *p* <.001, pseudo-R² = .11, with the Bayes factor providing overwhelming support. No further factors of avatar-age, avatar-gender or interactions improved model fit (all *p*s >.30) or increased pseudo-R² and Bayes factors provided moderate to extreme evidence in support of the angle-only model. See Table 6 for full results.

**Table 5.**

*Likelihood ratio test results and BIC derived Bayes factors for model comparisons with each step including a further independent variable or interaction term.*

| **Model** | **χ**2 | **Bayes factor** | ***p*** | **Pseudo R²** |
| --- | --- | --- | --- | --- |
| Intercept only |  |  |  |  |
| Comparison: Intercept  Angle | 75.54 | 965303109870 | <0.001 | .09 |
| Comparison: Intercept  Age | 0.01 | 0.01 | 0.90 | <0.001 |
| Comparison: Intercept  Gender | 3.77 | 0.04 | 0.05 | 0.001 |
| Comparison: Intercept  Age * Gender | 4.15 | 1.87e-6 | 0.25 | 0.001 |
| Comparison: Angle  Angle * Gender | 4.07 | 1.80e-6 | 0.25 | .09 |
| Comparison: Angle  Angle * Age | 2.17 | 6.96e-7 | 0.54 | .09 |
| Comparison: Angle  Angle * Age * Gender | 6.69 | 3.68e-19 | 0.67 | .09 |
| **Random Effects** | **χ**2 | **Bayes factor** | ***p*** | **Pseudo R²** |
| Comparison: Logistic regression without random effects  + Participant | 552.25 | 1.95e+122 | <0.001 |  |
| + Item  + Participant + Item | 2.55  556.65 | 45.18  3.51+121 | 0.11  <0.001 |  |
| Total residual |  |  |  | .40 |

**Table 6.**

*Results from model comparisons to assess the best fit for the observed data.*

| **Model** | **χ**2 | **Bayes factor** | ***p*** | **Pseudo R²** |
| --- | --- | --- | --- | --- |
| Intercept only |  |  |  |  |
| Comparison: Intercept  Angle | 117.18 | 1.12e+26 | <.001 | .11 |
| Comparison: Intercept  Age | 0.09 | <0.01 | 0.76 | <0.001 |
| Comparison: Intercept  Gender | <0.01 | <0.01 | 0.92 | <0.001 |
| Comparison: Intercept  Age * Gender | 2.54 | 9.05e-07 | 0.47 | <0.001 |
| Comparison: Angle  Angle * Gender | 0.56 | 3.36e-07 | 0.91 | .11 |
| Comparison: Angle  Angle * Age | 1.18 | 4.58e-07 | 0.76 | .11 |
| Comparison: Angle  Angle * Age * Gender | 7.85 | 8.27e-19 | 0.55 | .11 |
| **Random Effects** | **χ**2 | **Bayes factor** | ***p*** | **Pseudo R²** |
| Comparison: Linear model without random effects  + Participant | 9408.40 | Inf | <0.001 |  |
| + Item  + Participant + Item | 141.95  9660.30 | 4.22e+33  Inf | <0.001  <0.001 |  |
| Total residual |  |  |  | .41 |

1. The inclusion of age as a random effect produced models that did not converge under any optimizers. [↑](#footnote-ref-1)
